# DNA methylation profiling reveals a pathological signature that contributes to transcriptional defects of CD34^+^ CD15^−^ cells in early chronic‐phase chronic myeloid leukemia

**DOI:** 10.1002/1878-0261.12191

**Published:** 2018-04-27

**Authors:** Stéphanie Maupetit‐Mehouas, Franck Court, Céline Bourgne, Agnès Guerci‐Bresler, Pascale Cony‐Makhoul, Hyacinthe Johnson, Gabriel Etienne, Philippe Rousselot, Denis Guyotat, Alexandre Janel, Eric Hermet, Sandrine Saugues, Juliette Berger, Philippe Arnaud, Marc G. Berger

**Affiliations:** ^1^ GReD Université Clermont Auvergne CNRS INSERM Clermont‐Ferrand France; ^2^ Hématologie Biologique CHU Clermont‐Ferrand Hôpital Estaing Clermont‐Ferrand Cedex 1 France; ^3^ Equipe d'Accueil 7453 CHELTER Université Clermont Auvergne CHU Clermont‐Ferrand Hôpital Estaing Clermont‐Ferrand Cedex 1 France; ^4^ Hématologie Clinique CHRU Nancy Hôpitaux de Brabois Vandoeuvre‐lès‐Nancy France; ^5^ Hématologie Clinique CH Annecy‐Genevois Epagny Metz‐Tessy France; ^6^ Institut d'Hématologie de Basse Normandie CHU de Caen Caen Cedex 9 France; ^7^ Hématologie Clinique Institut Bergonié Bordeaux Cedex France; ^8^ Centre Hospitalier de Versailles service d'Hématologie et d'Oncologie Le Chesney France; ^9^ Département d'Hématologie Institut de Cancérologie Lucien Neuwirth Saint‐Priest‐en‐Jarez France; ^10^ Hématologie Clinique Adulte CHU Clermont‐Ferrand Hôpital Estaing Clermont‐Ferrand Cedex 1 France; ^11^ CRB‐Auvergne CHU Clermont‐Ferrand Hôpital Estaing Clermont‐Ferrand Cedex 1 France

**Keywords:** CML, DNA methylation, epigenetics, leukemic stem cells, transcriptional defects

## Abstract

Despite the high efficiency of tyrosine kinase inhibitors (TKI), some patients with chronic myeloid leukemia (CML) will display residual disease that can become resistant to treatment, indicating intraclonal heterogeneity in chronic‐phase CML (CP‐CML). To determine the basis of this heterogeneity, we conducted the first exhaustive characterization of the DNA methylation pattern of sorted CP‐CML CD34^+^CD15^−^ (immature) and CD34^−^CD15^+^ (mature) cells at diagnosis (prior to any treatment) and compared it to that of CD34^+^CD15^−^ and CD34^−^CD15^+^ cells isolated from healthy donors (HD). In both cell types, we identified several hundreds of differentially methylated regions (DMRs) showing DNA methylation changes between CP‐CML and HD samples, with only a subset of them in common between CD34^+^CD15^−^ and CD34^−^CD15^+^ cells. This suggested DNA methylation variability within the same CML clone. We also identified 70 genes that could be aberrantly repressed upon hypermethylation and 171 genes that could be aberrantly expressed upon hypomethylation of some of these DMRs in CP‐CML cells, among which 18 and 81, respectively, were in CP‐CML CD34^+^CD15^−^ cells only. We then validated the DNA methylation and expression defects of selected candidate genes. Specifically, we identified *GAS2*, a candidate oncogene, as a new example of gene the hypomethylation of which is associated with robust overexpression in CP‐CML cells. Altogether, we demonstrated that DNA methylation abnormalities exist at early stages of CML and can affect the transcriptional landscape of malignant cells. These observations could lead to the development of combination treatments with epigenetic drugs and TKI for CP‐CML.

**Abbreviation**
CGI: CpG island
CIMP: CpG‐island methylator phenotype
DMR: differentially methylated region
Ph: Philadelphia chromosome
TKI: tyrosine kinase inhibitor

## 1 Introduction

Chronic myeloid leukemia (CML) is one of the few examples of malignant transformation attributed to a single event, the translocation t(9; 22)(q34;q11) leading to the fusion of the *ABL* and *BCR* genes and known as Philadelphia chromosome (Ph). The resulting hybrid gene produces BCR‐ABL1, a chimeric oncoprotein with constitutive tyrosine kinase activity that promotes CML by aberrantly phosphorylating target proteins. Targeted treatments based on tyrosine kinase inhibitors (TKI) have shown considerable therapeutic effect (Gambacorti‐Passerini *et al*., 2011; Hochhaus *et al*., 2017).

However, despite the high efficiency of TKI‐based approaches, different observations highlight the intraclonal heterogeneity of chronic‐phase CML (CP‐CML) and the post‐treatment survival of a CML cell subpopulation. Specifically, residual disease can be detected even after treatment for several years (Hochhaus *et al*., 2017), and BCR‐ABL‐expressing leukemic stem cells (LSCs) can be observed in the bone marrow (BM) of patients with undetectable molecular residual disease. Moreover, CML relapses, due to the survival of a small number of LSCs in the BM (Chomel *et al*., 2016), are observed in about half of patients after treatment discontinuation (Etienne *et al*., 2017). In the case of secondary resistance to TKI, partial efficacy was reported before CML relapse due to a selected subclone (Druker, 2006).

The mechanisms of *in vivo* persistence of CML subclone(s) remain poorly understood. In the clinic, investigations have focused mainly on the occurrence of a *BCR‐ABL1* mutation and insufficient plasma level of TKI. However, most cases of CP‐CML resistance are not explained by these two situations (Cortes *et al*., 2009; Soverini *et al*., 2006). Besides genetic alterations, epigenetic deregulations and more specifically abnormal DNA methylation patterns also are attractive mechanisms that could contribute to explain LSC survival. Indeed, aberrant DNA methylation pattern is a hallmark of cancer (Kim and Costello, 2017). Specifically, cancer cells are characterized by genomewide DNA hypomethylation and hypermethylation of CpG islands (CGIs). CGIs are key regulatory regions that are often localized in promoter areas and that are constitutively unmethylated in normal cells.

DNA methylation also plays an important role in normal hematopoiesis and controls the fate of hematopoietic stem cells (HSC). For instance, deletion or functional loss of DNA (cytosine‐5)‐methyltransferase 1 (DNMT1) in murine models alters HSC self‐renewal and leads to their progressive loss (Trowbridge *et al*., 2009). The loss of the *de novo* methyltransferases DNMT3a and 3b favors HSC self‐renewal and blocks their differentiation (Challen *et al*., 2014). Furthermore, the TET enzymes, which are involved in the active DNA demethylation process, also influence cellular functions essential for HSC homeostasis (Guillamot *et al*., 2016).

However, DNA methylation alterations in CML and specifically in LSCs are poorly characterized. There are only few studies on DNA methylation alterations in CML and most of them focused on a small number of genes (Dunwell *et al*., 2010; Janssen *et al*., 2010; Jelinek *et al*., 2011; Qian *et al*., 2009; Strathdee *et al*., 2007; Sun *et al*., 2001; Uehara *et al*., 2012). They show that DNA methylation abnormalities increase with CML progression toward the accelerated phase and blast crisis (Dunwell *et al*., 2010; Janssen *et al*., 2010; Jelinek *et al*., 2011; Uehara *et al*., 2012). DNA methylation changes during CML progression have been confirmed by the only genomewide DNA methylation analysis (to our knowledge) of CML samples (Heller *et al*., 2016). However, this study is only partially informative, because it was carried out using mononuclear cells isolated from PB (PB) and BM. On the other hand, to understand the molecular basis of intraclonal heterogeneity, large‐scale DNA methylation analyses should be performed using defined CML clone subsets, including some enriched for LSCs. Currently, the existence and extent of DNA methylation alterations in these key cell populations and the DNA methylation profile differences in these various cell populations from the same CP‐CML clone remain to be determined.

To address this key issue, we carried out a large methylome analysis using sorted CD34^+^CD15^−^ (immature) and CD34^−^CD15^+^ (mature) cells from patients with CP‐CML at diagnosis, before any treatment and from healthy donors (HD). This allowed identifying specific disease‐related DNA methylation abnormalities and taking into account the methylation profile changes linked to cell differentiation (Bocker *et al*., 2011).

Our results provide the first exhaustive characterization of the DNA methylation pattern in CP‐CML, confirm the intraclonal heterogeneity of DNA methylation profiles, and suggest a functional impact of these methylation alterations on gene expression.

## 2 Methods

### 2.1 Patients, healthy donors, and human primary cells

A total of 13 patients with chronic‐phase CML (9M/4F; median age: 48 years (range: 24–90); Sokal score: 5 low, 5 intermediate, 3 high) (Table S1) were enrolled in the study after obtaining their written informed consent. This study was approved by the local ethics committee on human experimentation. Blood samples were collected in EDTA tubes at diagnosis, before any exposure to TKIs or any other treatment, and sent to the Clermont‐Ferrand center. All the sorting experiments were carried out with fresh cells, within 24 h of sampling.

To obtain the normal equivalent of circulating CML cells, we chose to use the leftovers of the samples used to evaluate the quality of G‐CSF‐mobilized peripheral blood progenitor cells (PBPCs) collected by apheresis from healthy donors (HD) [*n *=* *10; 6 M/4 F; median age: 34 years (range: 13–67)]. These samples could be used for research because donors were informed and did not verbally express any disagreement, as stipulated by French law.

### 2.2 Flow cytometry

All flow cytometry experiments were carried out using a BD FACS ARIA‐SORP flow cytometer equipped with five lasers (BD Biosciences, Le Pont de Claix, France). BD FACSDivaTM CS&T Research calibrated beads were used to track the cytometer performance each day and to generate reproducible data. Compensation settings were performed using BD CompBeads and the automatic compensation setup tool in the bd facs diva 7.0 software (BD Biosciences, Le Pont de Claix, France).

To sort target cell subsets, nucleated cells from patients with CP‐CML were isolated by collecting the buffy coat, followed by erythrocyte lysis using ammonium chloride (Stemcell Technologies Inc., Vancouver, Canada). PBPCs were directly used.

Briefly, 100–150 × 10^6^ cells were incubated in the dark with antibodies against 7‐AAD, CD45‐V500, CD34‐PC7, and CD15‐PerCpCy5.5 (BD Biosciences, Le Pont de Claix, France) for 20 min. Samples were then washed twice with PBS‐1% human AB serum and resuspended in PBS. Cells were sorted in two fractions, based on CD15 and CD34 markers to distinguish differentiated from undifferentiated cell subset: 7‐AAD^−^/CD45^+^/CD34^−^/CD15^+^ (CD34^+^CD15^−^ subset) and 7‐AAD^−^/CD45^+^/CD34^+^/CD15^−^ (CD34^−^CD15^+^ subset). Note that because of an overlap of expression between the CD34^+^ and the CD15^+^ populations (Fig. S1B), we defined the CD34^+^CD15^−^ and CD34^−^CD15^+^ subsets as cell subpopulations of interest and we excluded the ‘gray area’ of cells coexpressing both antigens.

The gating strategy is shown in Fig. S1. Side scatter (SSC) and forward scatter (FSC) parameters were used to eliminate cell doublets. Apoptotic (7‐AAD^+^) cells were excluded.

### 2.3 DNA extraction

DNA was extracted from CD34^+^CD15^−^ and CD34^−^CD15^+^ cells using the Nucleospin Tissue XS Kit (Macherey Nagel) following the manufacturer's protocol.

### 2.4 HM450K DNA methylation analyses

The HM450K array allows interrogating individually more than 485 000 CpG sites per sample, distributed in 99% of the annotated genes of the human genome. DNA bisulfite conversion and array hybridization were performed by Integragen, SA (Evry, France), using the Illumina Infinium HD methylation protocol. β‐values were normalized using the GenomeStudio control interplate normalization and background subtraction (version 2011.1). For each sample, β‐values with a detection *P*‐value >0.01 were excluded. All probes with a detection *P*‐value >0.01 or lacking signal in more than 5% of our cohort were rejected for the analysis. Finally, 26 957 probes containing common SNPs (dbSNP 138) in their last 5 bp or in the CpG sites were discarded. As our patient cohort included both men and women, probes on the X and Y chromosomes were also removed for the analysis.

Following above‐described quality filters, a total of 443 857 CpG methylation values were considered suitable for the downstream analysis. Differential methylation analyses were performed using the limma R package (Court *et al*., 2014). HM450K probes were considered differentially methylated when FDR was <0.05 and when the β‐value difference between groups was >0.15.

The β‐values for human embryonic stem cells (hESC), hematopoietic cells, acute myeloid leukemia (AML) CD34^+^CD38^−^ cells, and a panel of solid cancer samples were extracted from publicly available methylation data, in details:

(a) hESC: GSM1589944, GSM1589945, GSM1589946 and GSM999379.
(b) Hematopoietic cells: GSE35069.
(c) Acute myeloid leukemia CD34^+^CD38^−^ cells and normal hematopoietic stem cells: GSE63409.

Methylation data for solid cancers and their matched normal controls were extracted from the TCGA database as described in Court and Arnaud (2017).

### 2.5 Gene‐specific bisulfite sequencing and combined bisulfite restriction analysis

Bisulfite conversion, PCR amplification, digestion for combined bisulfite restriction analysis (COBRA), cloning, and sequencing were performed as previously described (Arnaud *et al*., 2006). Details on the primers and restriction enzymes are in Table S2.

### 2.6 Annotation and data mining‐associated approaches

#### 2.6.1 Comparison of HM450K β‐values and whole‐genome bisulfite‐sequencing data for CD34^+^ CD15^−^ cells

Whole‐genome bisulfite‐sequencing (WGBS) coverage and methylation values for PB‐CD34^+^ cells were obtained from the GEO database (accession number GSM791828). Only the methylation values of CpG sites that were covered by at least 5 reads in the WGBS experiment were analyzed (25 027 707 CpGs retained). The mean β‐values of the five HD‐CD34^+^CD15^−^ controls (HM450K data) were used for comparison. A total of 409 155 methylation values were compared between WGBS and HM450K.

#### 2.6.2 CD34^+^ cell gene expression and chromatin state

RNA‐seq data of PB‐CD34^+^ cells were downloaded from the GEO database (GSM1256827). Fastq files were mapped to the hg19 genome using tophat2 (version 2.1.0). Reads were aligned with a gene annotation reference (Gencode Release 19). Gene expression level was estimated with the Cufflinks tools using Gencode gene annotations (release 19). The chromatin state of PB‐CD34^+^ cells was evaluated using Input (GSM537662, GSM621414, GSM621456, GSM621689), H3K4me3 (GSM537652, GSM621437, GSM621439, GSM621665), and H3K27me3 data (GSM537649, GSM621431, GSM621664, GSM669945), as described in Court and Arnaud (2017).

#### 2.6.3 Genomic features and annotations

Gene and transcript positions in Gencode release 19 in GTF format for RNA‐seq data analysis were downloaded from the Gencode Web site. The positions of genes, SNPs, and CpG islands were downloaded from UCSC: Gencode basic V19, Common SNPs (138), and CpG‐island tracks, respectively. For each gene, promoter regions were defined as TSS ± 1 kbp. Their classification into high‐CpG‐density promoters (HCP), intermediate‐CpG‐density promoters (ICP), and low‐CpG‐density promoters (LCP) was performed using criteria defined by (Weber *et al*., 2007). Enhancer positions were determined with human_permissive_enhancers_phase_1_and_2.bed from the Fantom5 project. Chromatin state in hESC was from Court and Arnaud (2017).

#### 2.6.4 Functional analyses and gene annotations

Gene enrichment analyses were conducted with the functional annotation tools in the DAVID 6.8 Web site. The tumor‐suppressor gene or oncogene lists were obtained from http://www.ongene.bioinfo-minzhao.org, http://www.cta.lncc.br, and http://www.uniprot.org with the keywords tumor suppressor (KW‐0043) or proto‐oncogene (KW‐0656).

### 2.7 RNA isolation and RT/PCR expression analysis

RNA was extracted using TRIzol (Fisher Scientific, Illkirch, France) according to the manufacturer's recommendations. After treatment with RQ1 RNase‐free DNase I (Promega, Charbonnières‐les‐Bains, France), RNA (100–250 ng) was reverse transcribed (RT) using the Superscript IV kit (Fisher Scientific) and random primers. Three independent experiments were conducted for each sample (except for the *WT1* and *PRAME* transcripts, where two independent experiments were conducted). For each RNA sample, one RT was without reverse transcriptase to detect undesired amplification from DNA contamination. Real‐time PCR analyses were performed using the SYBR Green mixture (Roche, Meylan, France) and a LightCycler^®^ 480II (Roche) apparatus. Primers and amplification conditions are summarized in Table S2.

The relative expression level was quantified as follows: E^−Ct(Transcript)^/geometrical mean(E^−Ct(HK genes)^), based on the −2ddCt methods (E: efficiency of amplification, Ct: cycle threshold, HK: housekeeping). The housekeeping genes *GUSB, SDHA,* and *HMBS* were used to normalize transcript expression. The presented data are the mean ± standard deviation of two or three independent experiments, each in duplicate.

### 2.8 Data accessibility

The HM450K DNA methylation data generated in this study have been submitted to the NCBI Gene Expression Omnibus (GEO; http://www.ncbi.nlm.nih.gov/geo/) under accession number GSE106600.

## 3 Results

### 3.1 Progressive hypomethylation of healthy donor CD34^+^CD15^−^ and CD34^−^CD15^+^ cells

We first characterized the DNA methylation pattern of CD34^+^CD15^−^ and CD34^−^CD15^+^ cells from five HDs using the HM450K array. After quality filtration, we could assign a β‐value comprised between 0 (i.e., unmethylated position) and 1 (i.e., fully methylated position) to 443 857 CpG sites for each sample. We then compared the DNA methylation data of HD CD34^+^CD15^−^ cells with those obtained by whole‐genome bisulfite sequencing of PB CD34^+^ (PB‐CD34^+^) cells (GSM791828) (Hodges *et al*., 2011). The good correlation (*R* ^2^ = 0.89) validated the quality of the data obtained with the HM450K array (Fig. S2A). The specificity of our sorting approach was further supported using publicly available HM450K profiles of eight hematological cell types, PB mononuclear cells, and whole blood. Based on their methylation profile, HD CD34^−^CD15^+^ cells clustered in the myeloid subgroup, while HD CD34^+^CD15^−^ cells formed an independent group from both myeloid and lymphoid cells (Fig. S2B).

**Figure 1:**
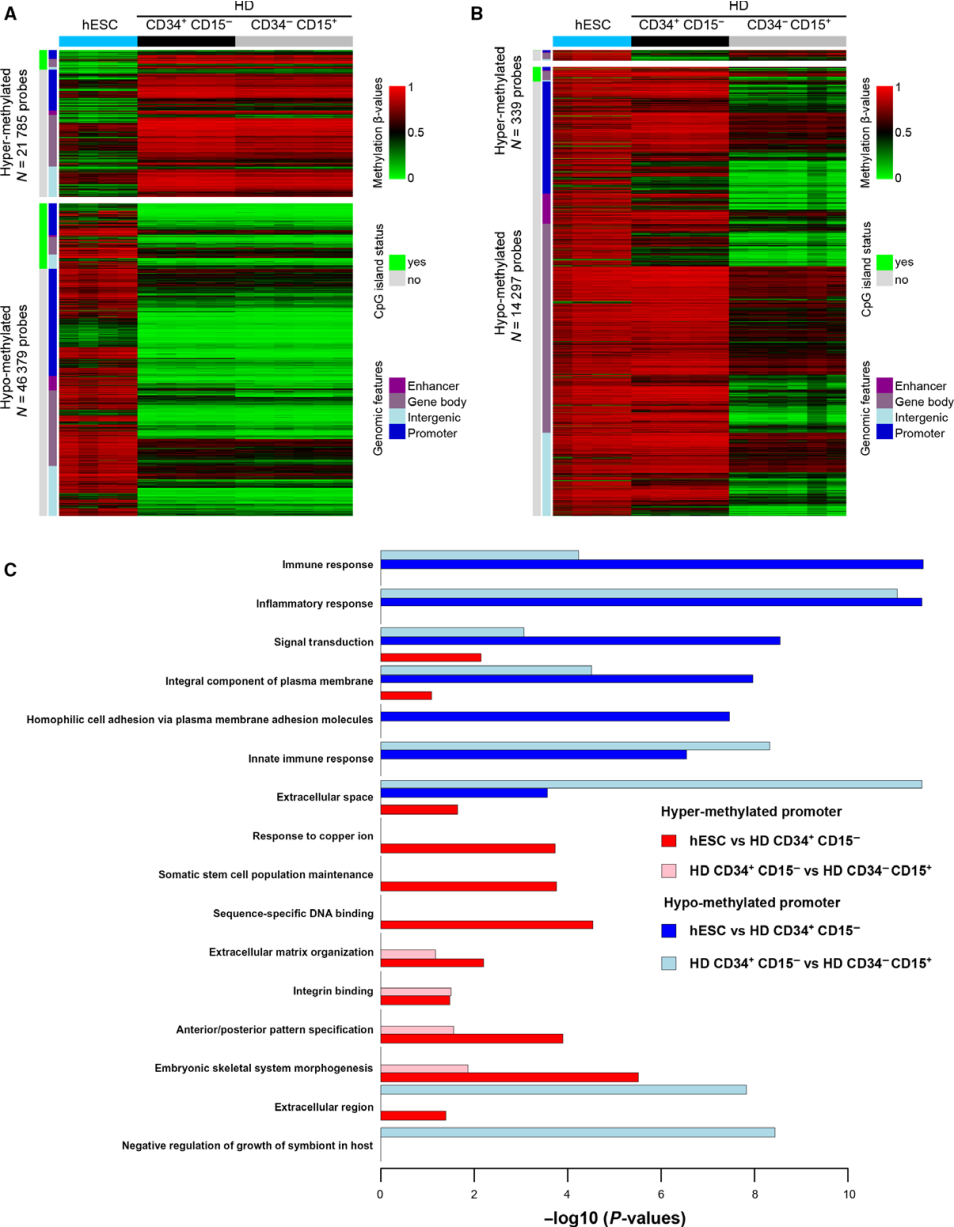
DNA methylation changes between hESCs, CD34^+^ CD15^−^, and CD34^−^ CD15^+^ cells from healthy donors. Heatmaps of differentially methylated probes between (A) hESC and HD CD34^+^ CD15^−^ cells and (B) HD CD34^+^ CD15^−^ and HD CD34^−^ CD15^+^ cells, respectively. The methylation level varies from low (β‐value close to 0; in green) to high (β‐value close to 1; in red). Hyper‐ and hypomethylated probes are shown on the upper and lower panels, respectively, and their number is indicated. The CpG‐island status and genomic features of the probes are indicated by color codes. (C) Gene ontology terms (GO database) in promoters with hyper‐ (red bars) and hypomethylated (blue bars) probes (n ≥ 2) between hESC and HD CD34 +  CD15‐ and between HD CD34^+^ CD15^−^ and HD CD34^−^ CD15^+^ cells.

We then evaluated changes in DNA methylation patterns during blood cell differentiation, from hESC to CD34^+^CD15^−^ hematopoietic stem/progenitor cells and finally to differentiated CD34^−^CD15^+^ cells. Most changes were between hESC and HD CD34^+^CD15^−^ cells (68 164 differentially methylated probes). Conversely, differentially methylated probes were five times less numerous between CD34^+^CD15^−^ and CD34^−^CD15^+^ cells. These changes were mainly associated with loss of DNA methylation. Specifically, 68% (46 379/68 164) of differentially methylated probes were hypomethylated in CD34^+^CD15^−^ cells compared with hESC. This trend was further accentuated upon hematopoietic differentiation, because 98% of DNA methylation changes between CD34^+^CD15^−^ and CD34^−^CD15^+^ cells corresponded to hypomethylation events (Fig. 1A,B). Similarly, myeloid cell types were globally unmethylated compared with HD CD34^+^CD15^−^ cells (Fig. S2C). These changes were underrepresented in CGIs (Fig. S2D), whereas they were widespread throughout the genome, including promoter and enhancer regions (Fig. 1A,B). Ontology analyses revealed that inflammatory and immune response genes were over‐represented among the genes with a hypomethylated promoter (*p* value < 10^−4^) (Figs 1C and S2E).

### 3.2 DNA methylation alterations in CP‐CML CD34^+^CD15^−^ and CD34^−^CD15^+^ cells

**Figure 2:**
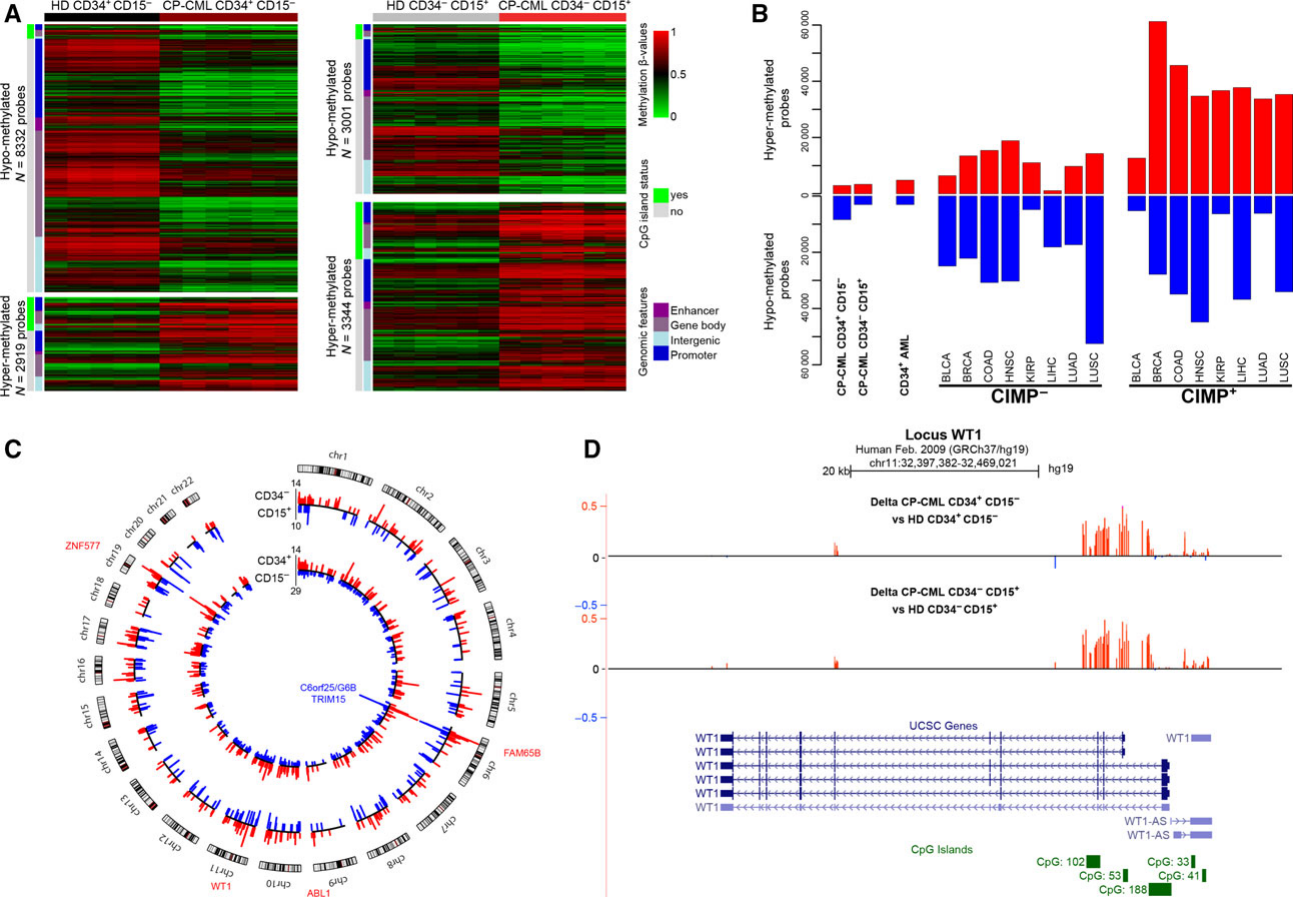
DNA methylation alterations in CP‐CML cells. (A) Heatmaps of differentially methylated probes in CP‐CML and HD CD34^+^ CD15^−^ (left panel) and CD34^−^ CD15^+^ (right panel) cells. Hypo‐ and hypermethylated probes are shown on the upper and lower panels, respectively, and their number is indicated. The CpG‐island status and genomic features of the probes are indicated by color codes. (B) Distribution of hyper‐ and hypomethylated probes in CP‐CML cells, AML CD34^+^ cells, and eight types of solid tumors. For each solid tumor type, CpG‐island methylator phenotype (CIMP)‐positive (+) and CIMP‐negative (−) samples were analyzed separately. BLCA, bladder urothelial carcinoma; BRCA, breast invasive carcinoma; COAD, colon adenocarcinoma; HNSC, head–neck squamous cell carcinoma; KIRP, kidney renal papillary cell carcinoma; LIHC, liver hepatocellular carcinoma; LUSC, lung squamous cell carcinoma; LUAD, lung adenocarcinoma. (C) Circular karyotype showing the position of hyper‐ (in red) and hypomethylated (in blue) DMRs in CP‐CML CD34^+^ CD15^−^ (inner circle) and CD34^−^ CD15^+^ (outer circle) cells. Some hotspot regions are named. (D) Detail of the *WT1* locus showing a cluster of hypermethylated probes in the two promoter regions in CP‐CML CD34^+^ CD15^−^ and CD34^−^ CD15^+^ cells compared with HD cells.

We next investigated the DNA methylation profiles of CD34^+^CD15^−^ and CD34^−^CD15^+^ cells from six patients with CP‐CML. Compared with HD cells, we observed DNA methylation alterations in both cell types. The extent of such defects was similar to what observed in AML cells, but more limited (up to 8 times) than in the representative panel of solid cancers, regardless of their CpG‐island methylator phenotype (CIMP) status (Fig. 2A,B).

We identified both hyper‐ and hypomethylated probes, with a majority of hypomethylation events (74%) in CP‐CML CD34^+^CD15^−^ cells. Overall, these changes were widespread throughout the genome and affected enhancer, promoter, gene body, and intergenic areas. In agreement with the hypomethylated status of CGIs in normal cells, most hypomethylation events observed in CP‐CML samples were in non‐CGI regions (Fig. 2A).

Specifically, 11 251 probes were differentially methylated in CP‐CML CD34^+^CD15^−^ cells and 6345 in CP‐CML CD34^−^CD15^+^ cells compared with the relevant HD samples. To further delineate the affected genomic loci, we mapped the regions that contained at least two consecutive hyper‐ or hypomethylated probes (absolute methylation difference >15%) in a maximal window of 1Kb between CP‐CML and the relevant HD cell samples. In CP‐CML CD34^+^CD15^−^ cells, we identified 1857 windows that could be merged into 400 hyper‐ and 742 hypomethylated differentially methylated regions (DMRs) of up to 2.3Kbp in size, respectively (Figs 2C, S3A and Table S3). In CP‐CML CD34^−^CD15^+^ cells, we identified 396 hyper‐ and 285 hypomethylated DMRs (Table S3). Only a subset of them (225 and 218, respectively) was in common with those identified in CD34^+^CD15^−^ cells, suggesting DNA methylation variability within the same CML clone (Fig. S3B). In both CP‐CML cell types, hypermethylated DMRs tended to be enriched at CGIs, specifically at CGIs located in the gene body, enhancer and intergenic areas, but not in promoter regions. Conversely, hypomethylated DMRs were enriched in enhancer and non‐CGI promoter regions, and more specifically in those with a low and, to a lesser extent, an intermediate‐CpG density (*i.e.,* LCP and ICP promoters (Weber *et al*., 2007), respectively) (Fig. S3C,D).

By considering regions with at least five consecutive affected probes, we defined 33 highly affected genomic areas in CP‐CML CD34^+^CD15^−^ cells (Table S4). Such dense clusters of hypermethylation included the translocated *ABL1* promoter, the promoter regions of Wilms tumor 1 (*WT1*), a transcription factor overexpressed in myeloid malignancies (Rosenfeld *et al*., 2003), and the promoter of the *ZNF577* gene that encodes a zinc finger protein (Figs 2C,D, S4A). On chromosome 6, hypomethylated clusters overlapped with the *G6B* (*C6orf25)* gene, a member of the immunoglobulin superfamily, and the promoter of *TRIM15* that encodes a focal adhesion protein (Figs 2C, S4A). Most of but not all these ‘hotspot’ areas were also present in CD34^−^CD15^+^ cells (Table S4). For example, some regions, including the *VARS, EGFL8,* and *EIF4E* promoters, were specifically hypomethylated only in CP‐CML CD34^+^CD15^−^ cells (Fig. S4B and Table S4).

Moreover, the 33 highly affected genomic areas found in CP‐CML CD34^+^CD15^−^ cells displayed methylation patterns different not only from those of HD CD34^+^CD15^−^ and CD34^−^CD15^+^ cells, but also from those of various hematological cell types and blood cells. This suggests that these methylation defects constitute a pathological signature (Fig. S4C).

### 3.3 The promoters of genes repressed in PB CD34^+^ cells are more prone to aberrant hypermethylation in CP‐CML CD34^+^CD15^−^ cells

Aberrant DNA hypermethylation of CGIs is a well‐defined feature of cancer cells. In agreement, about 58% of hypermethylated DMRs in CP‐CML CD34^+^CD15^−^ cells were localized in CGI regions.

**Figure 3:**
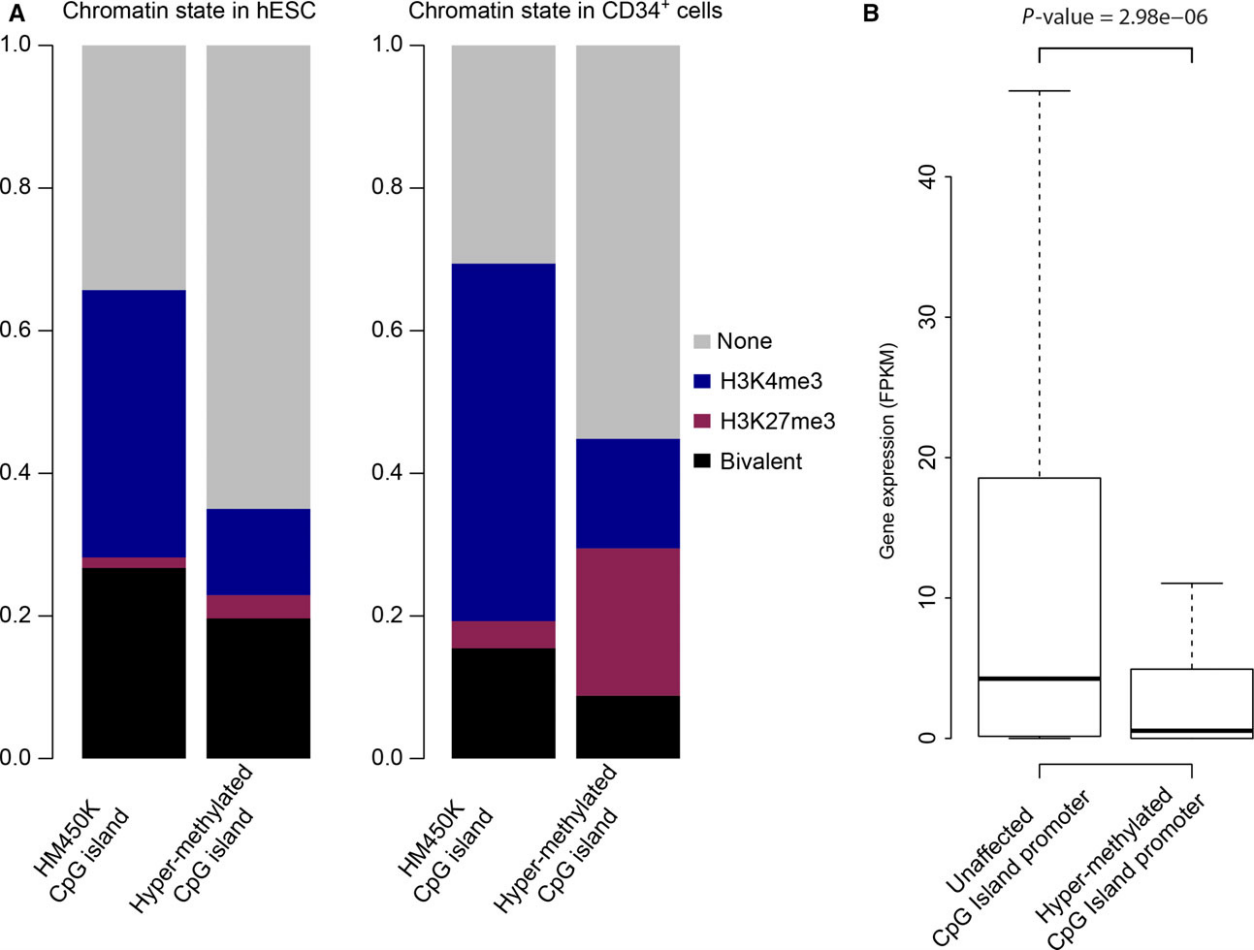
CpG island/promoters of genes repressed in PB CD34^+^ cells tend to be hypermethylated in CP‐CML CD34^+^ CD15^−^ cells. (A) Distribution of probes in hypermethylated CGI‐associated DMRs according to their chromatin signatures in hESCs (left panel) and PB (PB) CD34^+^ cells (right panel), respectively. The distribution of all HM450K probes at CGIs according to their chromatin signature in hESC and PB‐CD34^+^ cells, respectively, is shown as reference. (B) Genes with aberrantly hypermethylated CGI/promoter in CP‐CML CD34^+^ CD15^−^ cells tend to be repressed in PB‐CD34^+^ cells. Boxplot representation of the expression levels in PB‐CD34^+^ cells for genes with unaffected and hypermethylated CGI/promoter‐associated DMRs, respectively, in CML CD34^+^ CD15^−^ cells (Mann–Whitney test).

Studies on CGI hypermethylation in solid cancer and AML revealed that it is observed mainly at CGIs targeted by polycomb group proteins in embryonic or adult stem cells and particularly at CGIs that are concomitantly marked by the ‘active’ H3K4me3 and the ‘repressive’ polycomb‐derived H3K27me3 marks, constituting a bivalent chromatin signature (Court and Arnaud, 2017; Deneberg *et al*., 2011; Ohm *et al*., 2007). We did not observe this feature in CP‐CML CD34^+^CD15^−^ cells (Fig. 3A). CGIs with bivalent chromatin in hESC and in PB‐CD34^+^ cells were not preferentially hypermethylated in CP‐CML CD34^+^CD15^−^ cells. Rather, the 231 CGI‐associated hypermethylated DMRs were strongly enriched in CGIs depleted of both H3K4me3 and H3K27me3 in hESC or PB‐CD34^+^ cells (referred to as ‘non‐CGI’) and, to a lesser extent, in CGIs marked only by H3K27me3 in PB‐CD34^+^ cells. For instance, 65% and 55% of the probes localized at hypermethylated CGI‐associated DMRs were in non‐CGIs in hESC and PB‐CD34^+^ cells, respectively (compared with 34% and 30% of probes at non‐CGIs in the HM450K array).

Besides the chromatin signature in stem cells, the propensity of CGI/promoters to be aberrantly methylated in cancer cells has also been linked to the transcriptional activity in the matched normal tissues (Court and Arnaud, 2017; Sproul *et al*., 2011, 2012). We thus collected publicly available RNA sequencing data for PB‐CD34^+^ cells to determine whether the gene transcriptional status influenced the CGI/promoter methylation status in CP‐CML CD34^+^CD15^−^ cells. Overall, we observed a significantly lower expression in normal tissues of genes with promoter‐associated hypermethylated DMRs in tumor cells, independently of the presence/absence of CGIs in the promoter, compared with gene with unaltered DNA methylation pattern (Figs 3B, S5). This suggests that promoters of poorly expressed or repressed genes in healthy CD34^+^ cells are more likely to be aberrantly methylated in CML.

### 3.4 Lists of genes candidate to be affected by aberrant DNA methylation in CP‐CML CD34^+^CD15^−^ cells

**Figure 4:**
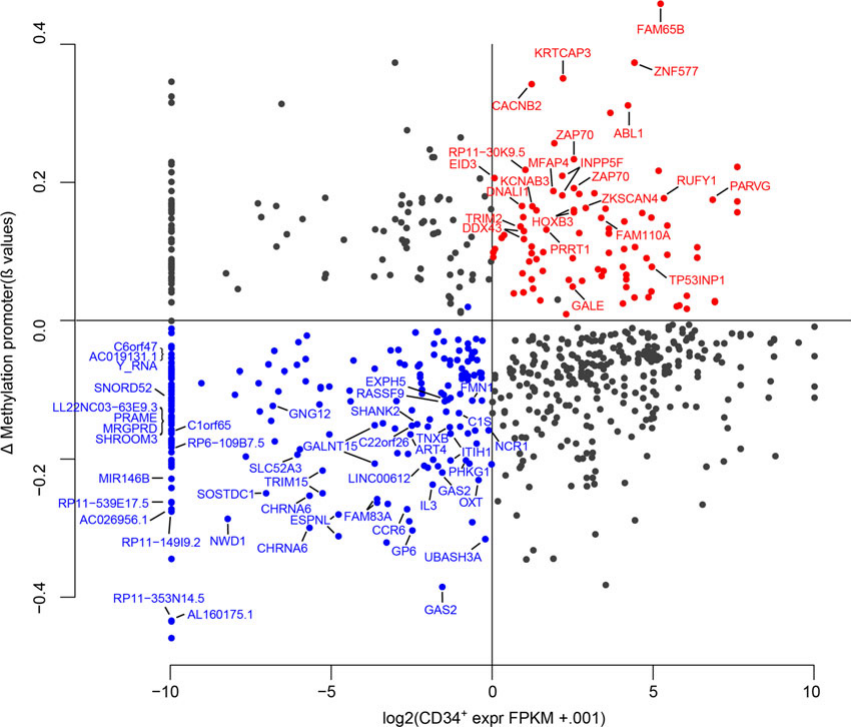
Candidate genes to aberrant expression following DNA methylation alterations in CP‐CML CD34^+^ CD15^−^ cells. Genes with DMRs in their promoter are classified according to the methylation variation between HD CD34^+^ CD15^−^ and CP‐CML CD34^+^ CD15^−^ cells (y‐axis) and level of expression in PB‐CD34^+^ cells (x‐axis). Blue and red dots represent genes that could be aberrantly expressed and repressed, respectively. Genes with four or more consecutive differentially methylated probes in their promoter are indicated.

The observation that hypermethylation mainly affects the promoter of genes that are already repressed in HD cells questions the impact of DNA methylation alterations on the transcriptional landscape of CP‐CML cells. To address this key point, and in the absence of publicly available RNA‐seq data for CML cells, we used RNA‐seq data from PB‐CD34^+^ cells to determine a list of candidate genes the expression of which could be repressed by aberrant DNA methylation in CP‐CML CD34^+^CD15^−^ cells (i.e., genes that are expressed in PB‐CD34^+^ cells (rpkm > 1) and the promoter of which is a hypermethylated DMR in CML). We found that 280 promoters of 177 genes (some genes have several promoters) were hypermethylated in CP‐CML CD34^+^CD15^−^ cells. Among them, 70 (39.5%) were expressed in PB‐CD34^+^ and consequently could be aberrantly repressed in CP‐CML CD34^+^CD15^−^ cells (red dots in Fig. 4; Table S5). They included the tumor‐suppressor *TP53INP1* (tumor protein P53‐inducible nuclear protein), *ZAP70* (zeta‐chain‐associated protein kinase 70) and *HOXB3*.

Besides focal CGI/promoter hypermethylation, cancer cells are also characterized by concomitant widespread DNA hypomethylation that contributes to genome instability and could lead to ectopic expression by affecting promoter activity. However, this last event is rare and documented for a limited number of genes, such as cancer‐germline genes (Van Tongelen *et al*., 2017) and inserted viral oncogenes. To determine whether some genes are reactivated following hypomethylation, we investigated genes that are repressed in PB‐CD34^+^ cells (rpkm < 1) and display hypomethylated DMRs in the promoter in CP‐CML CD34^+^CD15^−^ cells. Unexpectedly, this list included much more candidate genes than the one for genes with hypermethylated promoters. Among the 403 genes with hypomethylated promoters (*n* = 802) in CP‐CML cells, 171 (42.3%) could be aberrantly expressed (blue dots in Fig. 4; Table S5). This list included several oncogenes, namely *MIR544A*,* MIR146B*,* FAM83A* (also known as tumor antigen *BJ‐TSA‐9)*, the cancer‐germline genes *PBK* (PDZ binding kinase) and *PRAME* (preferentially expressed antigen in melanoma), and *GCKR* that, with *PRAME*, is a downstream target of the BCR‐ABL protein (Shi *et al*., 1999; Watari *et al*., 2000).

Except for the over‐representation for genes subject to alternatives splicing among hypermethylated genes (*P* value <10^−2^), no specific ontology term was associated with these two candidate gene lists (Fig. S6A).

To restrict the list to genes that could explain the specific features of CP‐CML CD34^+^CD15^−^ cells, we considered only promoters that were aberrantly methylated specifically in this cell subset and identified 81 and 18 genes that could be abnormally expressed and repressed, respectively, only in CP‐CML CD34^+^CD15^−^ cells (Fig. S6B and Table S6).

### 3.5 Methylation alterations can correlate with modified expression of candidate genes

To determine whether the expression of the genes in these two lists was affected in CP‐CML CD34^+^CD15^−^ cells and to validate the HM450K data in an independent cohort, we performed DNA methylation and expression analyses of selected genes using independent CP‐CML and HD CD34^+^CD15^−^ samples (*n *=* *7 and *n *=* *5, respectively).

**Figure 5:**
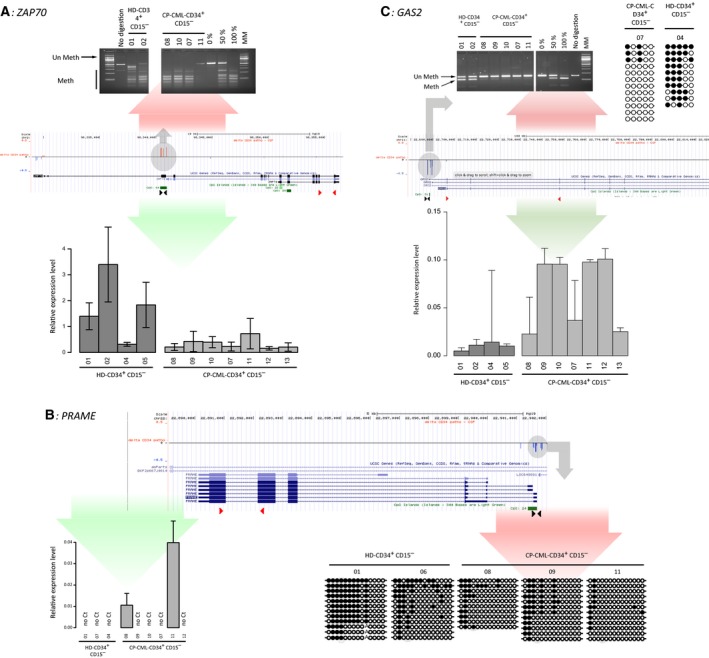
Gene expression and DNA methylation analyses at selected candidate genes. Results of the gene expression and DNA methylation analyses (COBRA) for the *ZAP70* (A), *PRAME* (B), and *GAS2* (C) genes in independent CP‐CML and HD cell samples. For each gene, the central panel indicates areas with differences in DNA methylation levels between CP‐CML and HD CD34^+^ CD15^−^ cells (HM450K array data). Red, methylation gain; blue, methylation decrease. The upper panel shows bisulfite‐based DNA methylation analyses by COBRA and/or sequencing in HD and CP‐CML samples. For each sequenced region, the methylation patterns are symbolized by lollipops (black: methylated CpG; white: unmethylated CpG). Expression data (quantitative PCR) are in the lower panel.

Expression data analysis identified *ZAP70* and *INPP5F‐V2* as genes that could be repressed by aberrant DNA methylation. The COBRA approach indicated that the CGI/promoter governing the main *ZAP70* isoform gained DNA methylation in CP‐CML samples, validating the HM450K data. This hypermethylation correlated with a decrease in gene expression in the same samples (Fig. 5A). The same analyses confirmed a gain of methylation on the CGI/promoter of the isoform‐V2 of the imprinted gene *INPP5F*, which encodes a phosphatidylinositide phosphatase, in CP‐CML samples. This gain correlated with aberrant *INPP5F* silencing (Fig. S7A).

We also observed a hotspot of aberrant hypermethylation at the promoter regions of *WT1* (Fig. 2C), a gene that is overexpressed in CML cells (Gerber *et al*., 2011; Rosenfeld *et al*., 2003). A more detailed analysis showed that this methylation gain affected the whole downstream alternative CpG island/promoter, but only the 3′ edge of the upstream CpG‐island promoter, while the TSS area remained free of methylation (Figs 2C, S7B). Accordingly, promoter‐specific RT/PCR analysis showed that *WT1* overexpression in CP‐CML CD34^+^CD15^−^ cells initiated mainly from the upstream hypomethylated CpG‐island promoter, because the transcript that initiates from the alternative hypermethylated promoter was not detected (Fig. S7B).

It is well documented that in cancer, promoter hypomethylation is associated with reactivation of cancer‐germline genes. In agreement, we observed that *PRAME* overexpression was correlated with its promoter hypomethylation, but only in a subset of CP‐CML samples (e.g., CP‐CML samples 8 and 11 but not CP‐CML sample 9) (Fig. 5B). Outside this class of genes, this phenomenon is a rare event. We then assessed the expression of *GAS2* (growth arrest‐specific 2) because it contributes to CML cell growth (Zhou *et al*., 2014) and thus could be an oncogene. COBRA and bisulfite‐sequencing approaches confirmed the results of the HM450K array data analysis: The *GAS2* promoter region was hypomethylated in all CP‐CML samples compared with HD cells. In all cases, this correlated with a robust, though variable, gain of *GAS2* expression in CP‐CML CD34^+^CD15^−^ cells (Fig. 5C).

## 4 Discussion

We report here the first exhaustive DNA methylation analysis of cell subsets from patients with CP‐CML at diagnosis before any treatment. To ensure the identification of DNA methylation differences only related to the pathology, we compared the DNA methylation profiles of immature CD34^+^CD15^−^ and mature CD34^−^CD15^+^ cells from patients with CP‐CML and HDs. Moreover, as the hematopoietic tissue origin could influence the cell methylation profile, we compared circulating CP‐CML cells and HD PBPCs that are believed to be equivalent to circulating CML cells (Farlik *et al*., 2016).

Our study confirmed and extended the results of previous works (Dunwell *et al*., 2010; Heller *et al*., 2016; Janssen *et al*., 2010; Jelinek *et al*., 2011; Qian *et al*., 2009; Strathdee *et al*., 2007; Sun *et al*., 2001; Uehara *et al*., 2012) showing that the DNA methylation landscape is altered at CP‐CML diagnosis. To better characterize these alterations, we identified the affected loci and DMRs. Only a subset of these regions were affected in both CD34^+^CD15^−^ and CD34^−^CD15^+^ cells, indicating that DNA methylation could contribute to the clonal heterogeneity of the early CML phase.

The DNA methylation defects at the 33 ‘strongest’ DMRs constitute a pathological signature and could be considered as candidate biomarkers of Ph clones. These DMRs included the *ABL1* and *WT1* genes the aberrant methylation of which has already been reported in chronic phase (Sun *et al*., 2001) and blast crisis (Janssen *et al*., 2010), respectively.

In immature CP‐CML CD34^+^CD15^−^ cells, we identified about two times more hypomethylated than hypermethylated DMRs (742 *vs* 400), suggesting that the trend toward hypomethylation observed during normal differentiation into the myeloid lineage (Farlik *et al*., 2016) is accentuated during malignant transformation. As observed in other cancers, hypermethylated DMRs are mainly localized in CGI regions. However, and unlike other cancers (Court and Arnaud, 2017; Deneberg *et al*., 2011; Ohm *et al*., 2007), the hypermethylation gain appears to be only partially predefined by a polycomb‐derived chromatin signature in healthy embryonic or adult stem cells. Although our study supports that CGIs marked by H3K27me3 alone in PB‐CD34^+^ are more prone to hypermethylation, it also highlights that CGIs with bivalent chromatin are not preferentially methylated in CP‐CML CD34^+^CD15^−^ cells. The reason for this discrepancy is unclear and could result from the limited number of hypermethylated DMRs we identified. It could also result from a protection brought by the TET‐derived 5 hydroxymethylcytosine (5hmC) modification. TET proteins, particularly TET2, are key regulators of human HSC biology (Langlois *et al*., 2014; Pronier *et al*., 2011). Specifically, by catalyzing the conversion of 5 methylcytosine (5mc) to 5hmC throughout the genome, TET2 might contribute to the marked trend toward hypomethylation during normal hematopoiesis (Tekpli *et al*., 2016). A recent study conducted in colorectal cancer revealed that promoters marked by 5hmC in normal tissue are resistant to DNA methylation gain in tumor cells. This study also highlighted that a relevant subset of these promoters overlaps with those marked by a bivalent signature in stem cells (Uribe‐Lewis *et al*., 2015). Therefore, high level of TET2‐mediated 5hmC in a subset of CGI/promoters with bivalent chromatin in healthy CD34^+^ cells could protect them against aberrant methylation in CP‐CML cells. In agreement, we observed that hypermethylation mainly affects nonpromoter CGIs in CP‐CML CD34^+^CD15^−^ cells, and CGI promoters in solid cancers (Court and Arnaud, 2017).

To which extent alterations in the DNA methylation landscape affect the transcriptional profile in CP‐CML CD34^+^CD15^−^ cells remains a pending question. To address this key question, and in the absence of publicly available RNA‐seq data on HD and CP‐CML CD34^+^CD15^−^ cells, we evaluated the transcriptional status of PB CD34^+^ cells to identify candidate genes the expression of which could be affected by aberrant DNA methylation of their promoter in CP‐CML CD34^+^CD15^−^ cells. In agreement with what observed in other cancers (Court and Arnaud, 2017; Sproul *et al*., 2011, 2012), DNA hypermethylation mainly affected the promoter of genes already repressed in healthy cells. This phenomenon probably restricts the overall functional impact of this defect. Not exclusively, this stable repression could also affect the tumor biology by limiting its epigenetic plasticity and, for instance, its ability to adapt following environmental changes, such as metastasis formation or treatment (Sproul and Meehan, 2013).

Nonetheless, we identified 70 genes that could be aberrantly repressed by DNA methylation in CP‐CML CD34^−^CD15^+^ cells and confirmed the repression of two of them in an independent cohort. Among these genes, *ZAP70* encodes a kinase essential for thymopoiesis, T‐cell receptor‐dependent survival, and proliferation (Schim van der Loeff *et al*., 2014) and is aberrantly expressed in B‐cell malignancies, such as acute lymphoblastic leukemia and chronic lymphocytic leukemia (Rassenti *et al*., 2004). This repression could be related to the preferential commitment to the myeloid lineage in CML.

DNA hypermethylation could also be associated with differential promoter usage or gene overexpression, as documented in prostate cancer (Bert *et al*., 2013). Although our strategy was not designed to identify such candidate genes, we observed that DNA hypermethylation of the inner WT1 CGI/promoter is associated with overexpression from the upstream promoter. A causal effect between these events remains to be evidenced.

Promoter hypomethylation leading to ectopic expression in cancer cells is a rare event. Therefore, the identification of 170 genes that could be aberrantly expressed in CP‐CML CD34^+^CD15^−^ cells was unexpected. In breast cancer, DNA methylation loss at promoters can be compensated by a gain of the repressive H3K27me3 mark (Hon *et al*., 2012); therefore, many of these candidate genes might remain repressed in cancer cells. For instance, we observed the maintenance of a DNA methylation‐independent repressed state at the *GP6* and *TRPV4* genes (data not shown). Nonetheless, our validation experiments and literature data indicate that several genes of this list are aberrantly expressed, for example *IL‐3* that is produced by CML cells and a therapeutic target (Frolova *et al*., 2014; Jiang *et al*., 1999). Similarly, the cancer‐germline gene *PRAME* is overexpressed in various cancer and leukemia types as well as in CML CD34^+^ cells (Gerber *et al*., 2011). Although it has already been shown that DNA hypomethylation at *PRAME* can lead to its overexpression (Schenk *et al*., 2007), the variable effect we observed in CP‐CML samples suggests that the decrease in DNA methylation at the *PRAME* promoter is not sufficient to induce ectopic expression. Among other candidates, *GAS2* deserves a specific attention. This gene is an indirect target of the BCR‐ABL chimeric protein (Hjort *et al*., 2016), and a candidate oncogene because of its involvement in CML cell growth and survival, particularly via the Wnt/β‐catenin pathway (Huang *et al*., 2017; Zhou *et al*., 2014). We confirmed in an independent cohort that *GAS2* is robustly hypomethylated and overexpressed in all the studied cell samples, thus emerging as one of the rare genes in which promoter hypomethylation is associated with expression reactivation in cancer cells.

Interestingly, it has been reported that *WT1*,* PRAME,* and *GAS2* are overexpressed during CML progression toward the blast crisis phase (Radich *et al*., 2006). This observation suggests that the subpopulation of cells in which these genes are aberrantly methylated is selected during CML progression.

## 5 Conclusions

In conclusion, we showed that DNA methylation abnormalities exist at early stages of CP‐CML and can affect the transcriptional profile of malignant cells. Although their role in TKI resistance and CML progression remains to be determined, these alterations emerge as relevant candidates for disease progression or resistance of subclones.

The characterization of DNA methylation alterations specifically in immature CD34^+^CD15^−^ cells and the identification of several candidate genes implicated in CML progenitor survival or proliferation open new avenues for developing combination therapies with epigenetic drugs and TKIs for the treatment of CP‐CML.

## Authors contributions

SMM, FC, CB, PA, and MGB designed the study and analyzed the data. PCM, HJ, GB, PR, DG, EH, AJ provided patient samples. CB performed the immunophenotyping, cell sorting, and DNA extraction. JB prepared primary samples and stored the sorted subpopulations in the CRB‐Auvergne, an NF96‐900‐labeled structure. SS collected the associated patients’ data and verified the presence of informed consents. SMM, FC, and CB performed the experiments and realized the figures. FC did the data mining and bioinformatics analyses. PA and MGB coordinated the study and wrote the manuscript.

## Supporting information


**Fig. S1.** CD34^+^CD15^−^ and CD34^−^CD15^+^ cells sorting.

**Fig. S2.** Characterization of the methylation profile of HD CD34^+^CD15^−^ and HD CD34^−^CD15^+^ cells.

**Fig. S3.** Characterization of DNA methylation alterations in CP‐CML cells.

**Fig. S4.** Details of the DMRs with affected methylation in CP‐CML cells.

**Fig. S5.** Promoters of genes repressed in PB CD34^+^ cells tend to be hypermethylated in CP‐CML CD34^+^CD15^−^ cells.

**Fig. S6.** Candidate genes to aberrant expression following DNA methylation alterations in CP‐CML CD34^+^CD15^−^ cells.

**Fig. S7.** INPP5F‐V2 repression and WT1 overexpression in CP‐CML cells**.**

**Table S1.** Details of the patients with CP‐CML.

**Table S2.** (XLSX): Details of the primers used in this study.

**Table S3.** (XLSX): List of DMRs identified in CP‐CML CD34^+^CD15^−^ and CP‐CML CD34^−^CD15^+^ cells, according their methylation status.

**Table S4.** Hotspots of DNA methylation alteration in CP‐CML cells. Genomic positions are according to hg19.

**Table S5.** (XLSX): List of candidate genes to aberrant repression upon DNA hypermethylation, or to aberrant expression following DNA hypomethylation in CP‐CML CD34^+^CD15^−^ cells.

**Table S6.** List of candidate genes to aberrant repression following DNA hypermethylation, or aberrant expression following DNA hypomethylation specifically in CP‐CML CD34^+^CD15^−^ cells.
