# Supplementary figures and images for: DNA methylation profiling reveals a pathological signature that contributes to transcriptional defects of CD34+ CD15− cells in early chronic‐phase chronic myeloid leukemia
Source: Mol Oncol. 2018 Apr 27;12(6):814–29. doi: 10.1002/1878-0261.12191 (PMC5983208; doi:10.1002/1878-0261.12191)

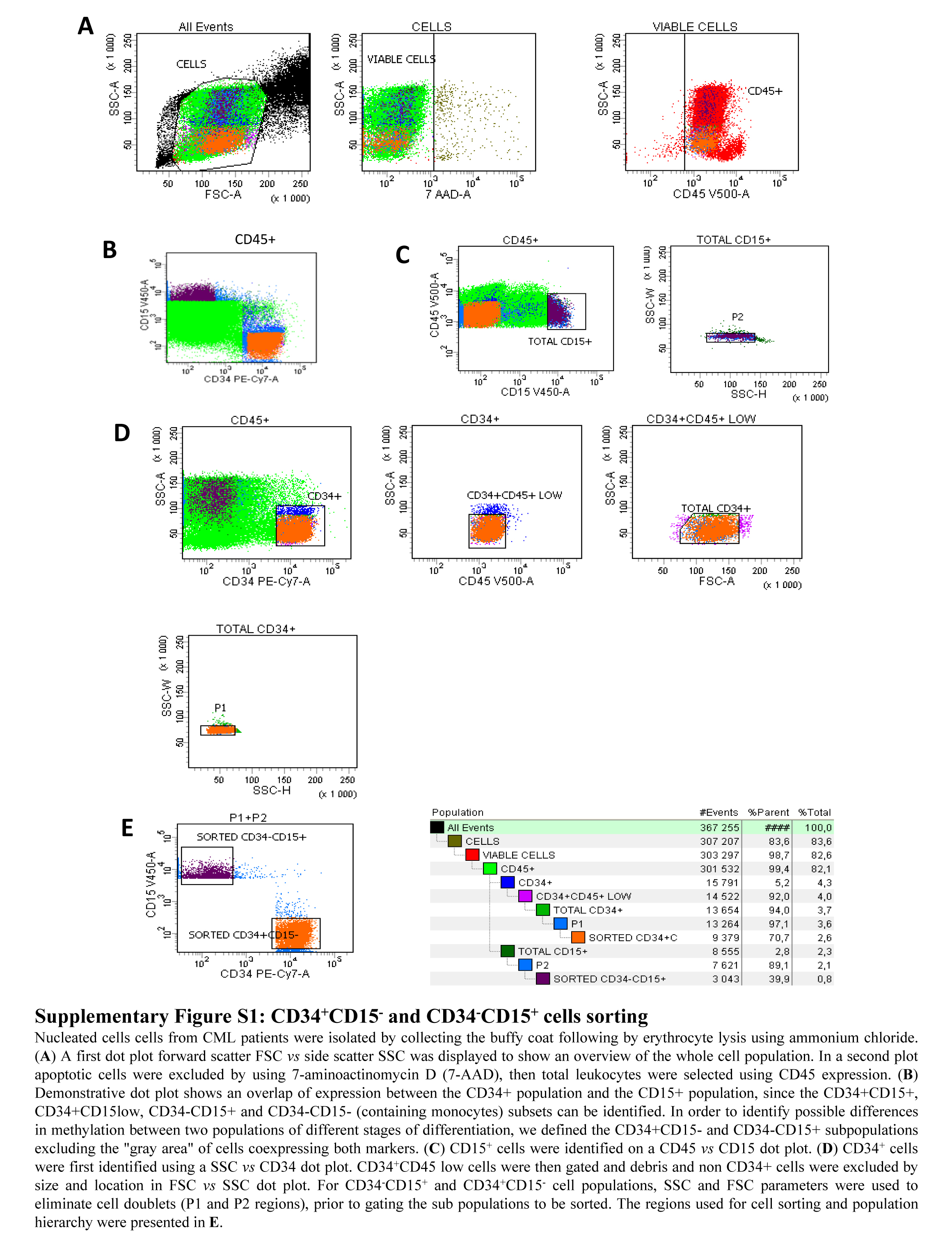

Supplement: Supplementary file 1 — Fig. S1. CD34+CD15− and CD34−CD15+ cells sorting. [file MOL2-12-814-s001.jpg]

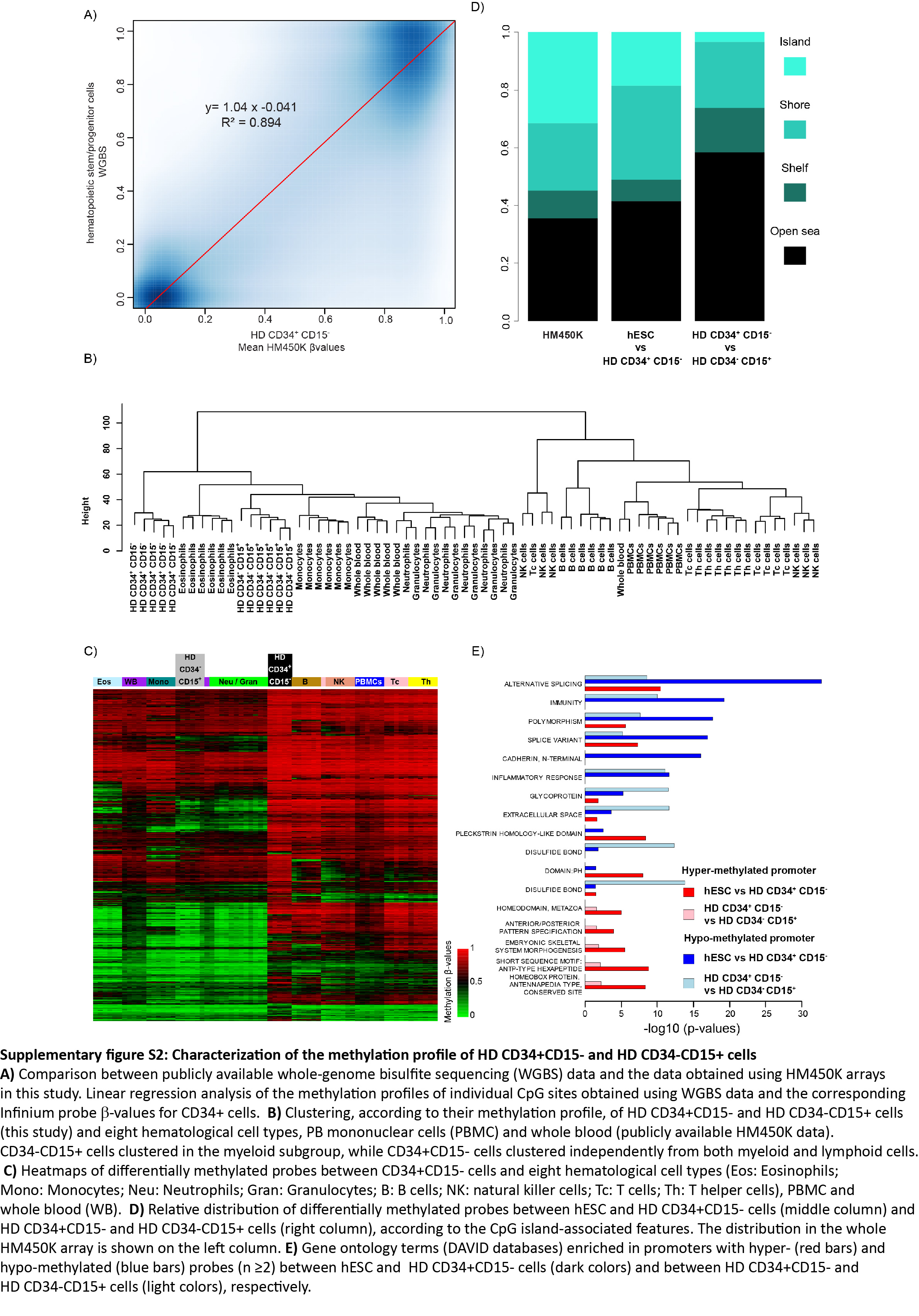

Supplement: Supplementary file 2 — Fig. S2. Characterization of the methylation profile of HD CD34+CD15− and HD CD34−CD15+ cells. [file MOL2-12-814-s002.jpg]

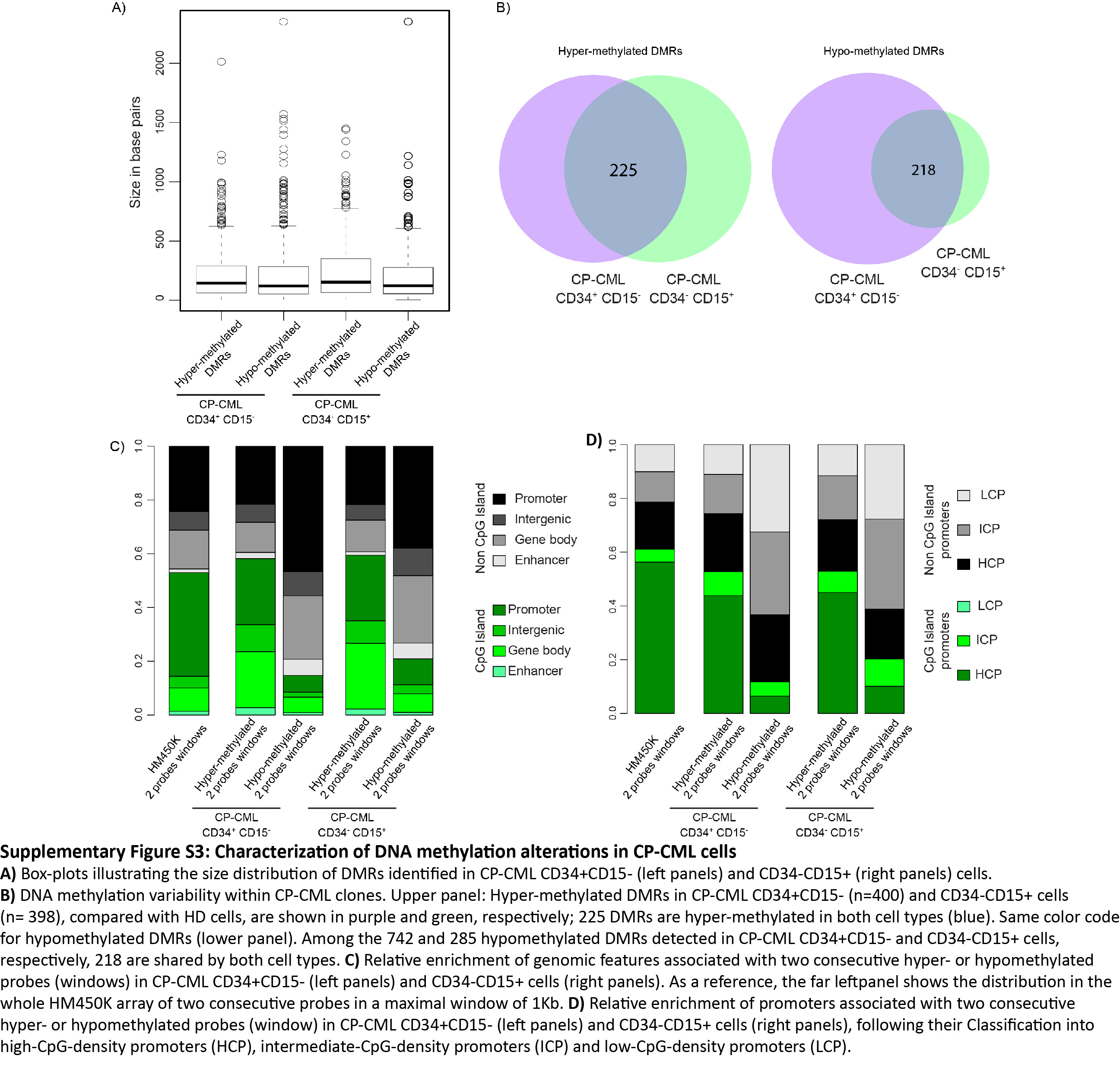

Supplement: Supplementary file 3 — Fig. S3. Characterization of DNA methylation alterations in CP‐CML cells. [file MOL2-12-814-s003.jpg]

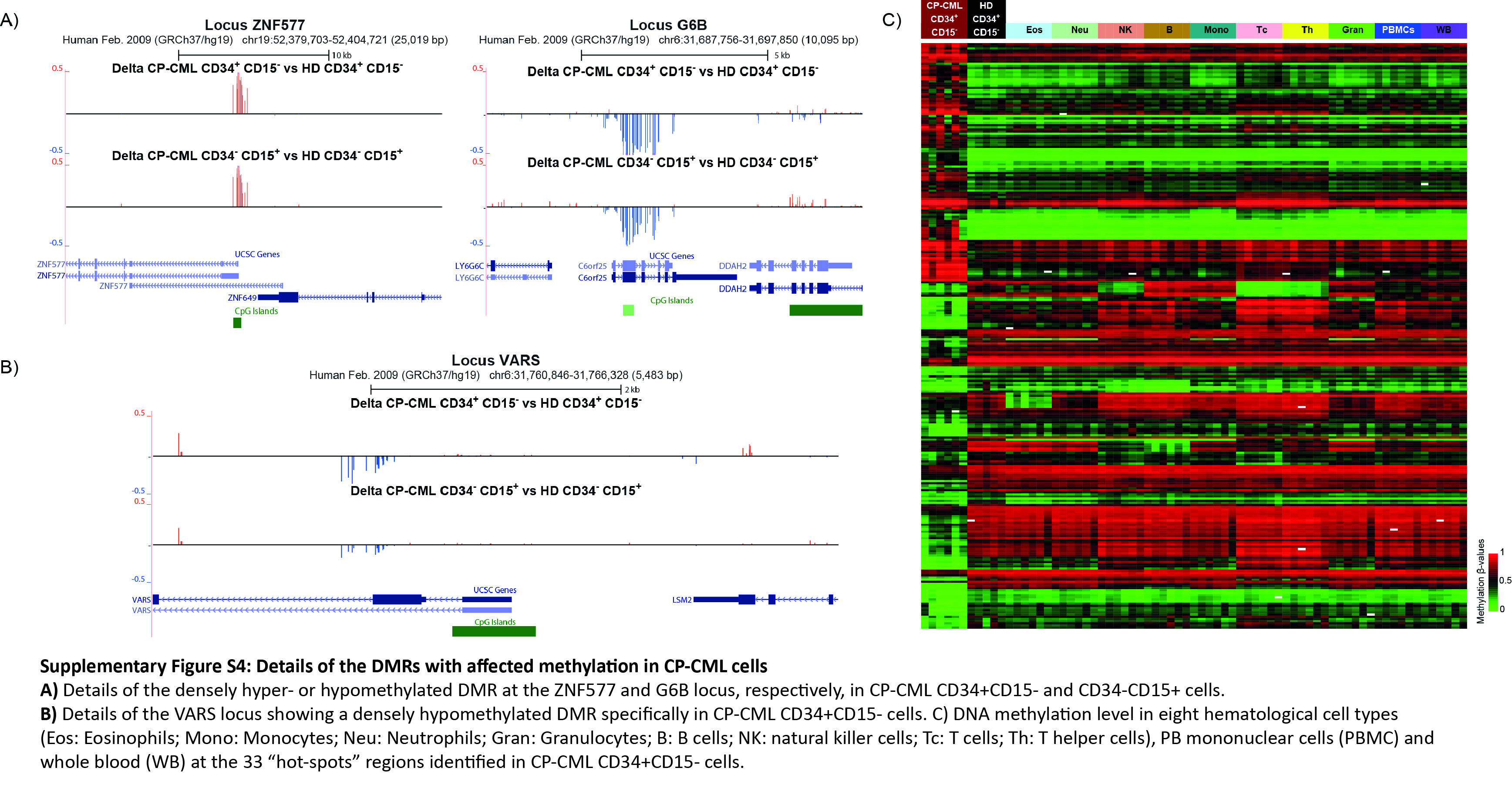

Supplement: Supplementary file 4 — Fig. S4. Details of the DMRs with affected methylation in CP‐CML cells. [file MOL2-12-814-s004.jpg]

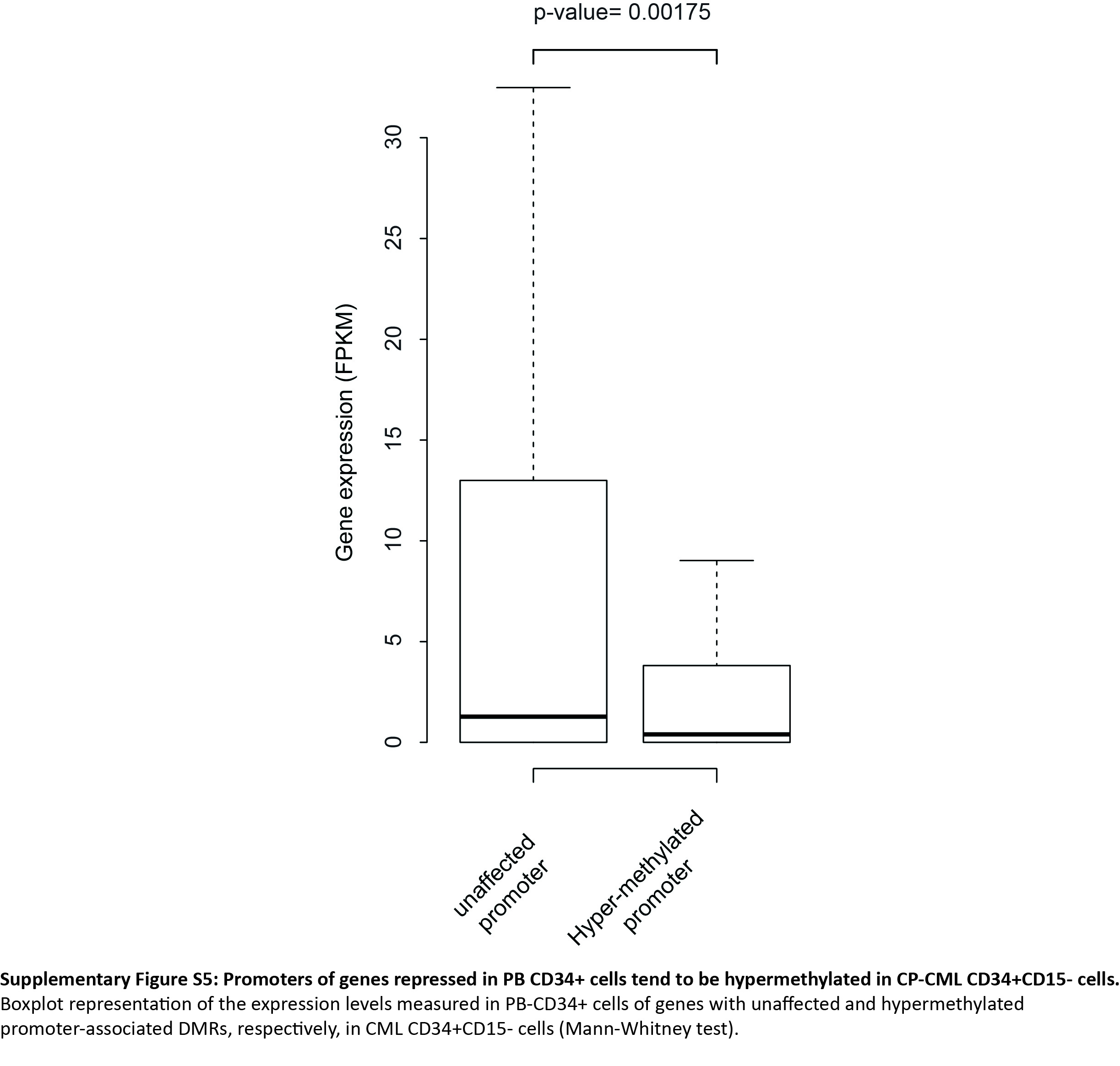

Supplement: Supplementary file 5 — Fig. S5. Promoters of genes repressed in PB CD34+ cells tend to be hypermethylated in CP‐CML CD34+CD15− cells. [file MOL2-12-814-s005.jpg]

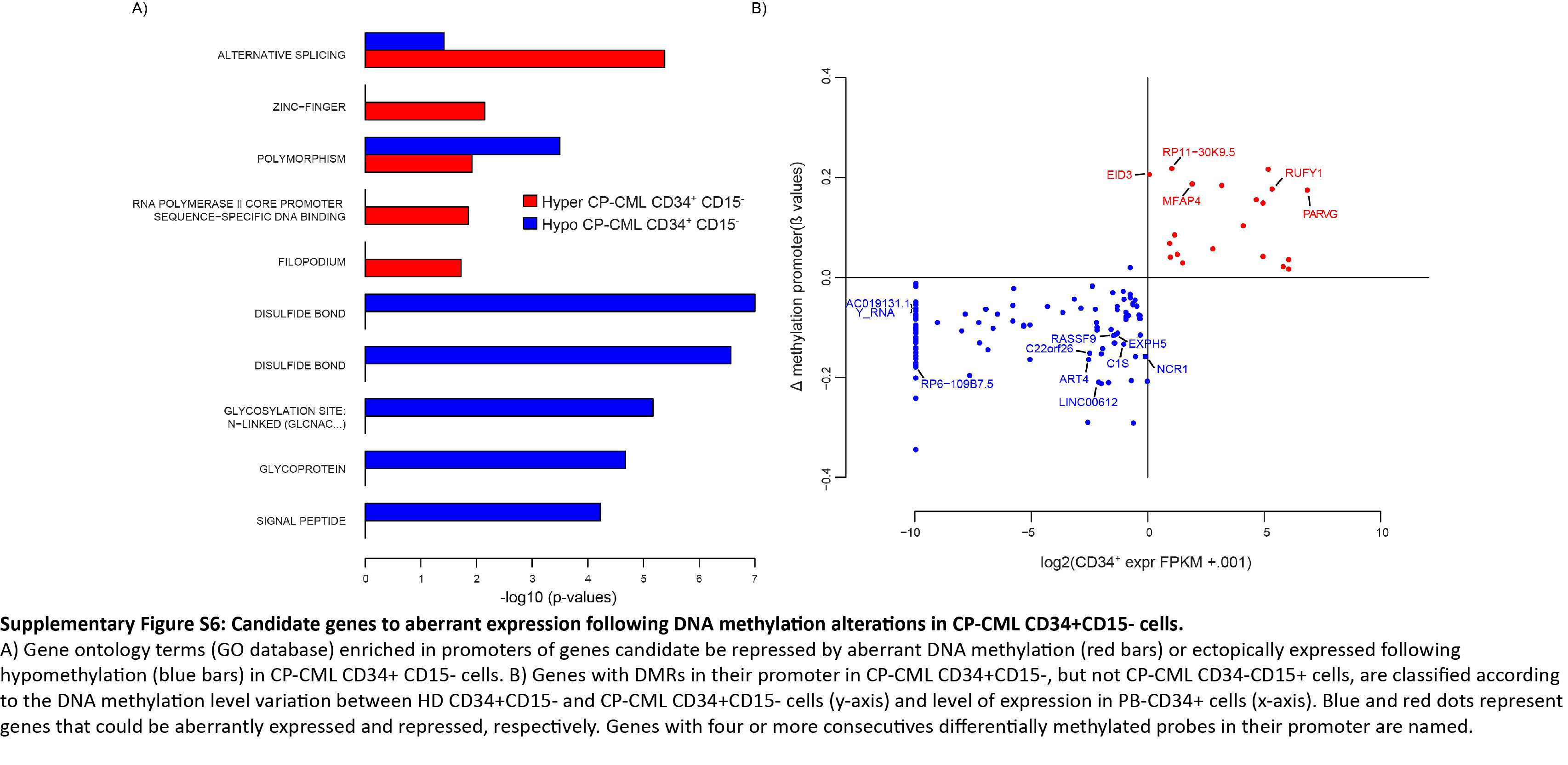

Supplement: Supplementary file 6 — Fig. S6. Candidate genes to aberrant expression following DNA methylation alterations in CP‐CML CD34+CD15− cells. [file MOL2-12-814-s006.jpg]

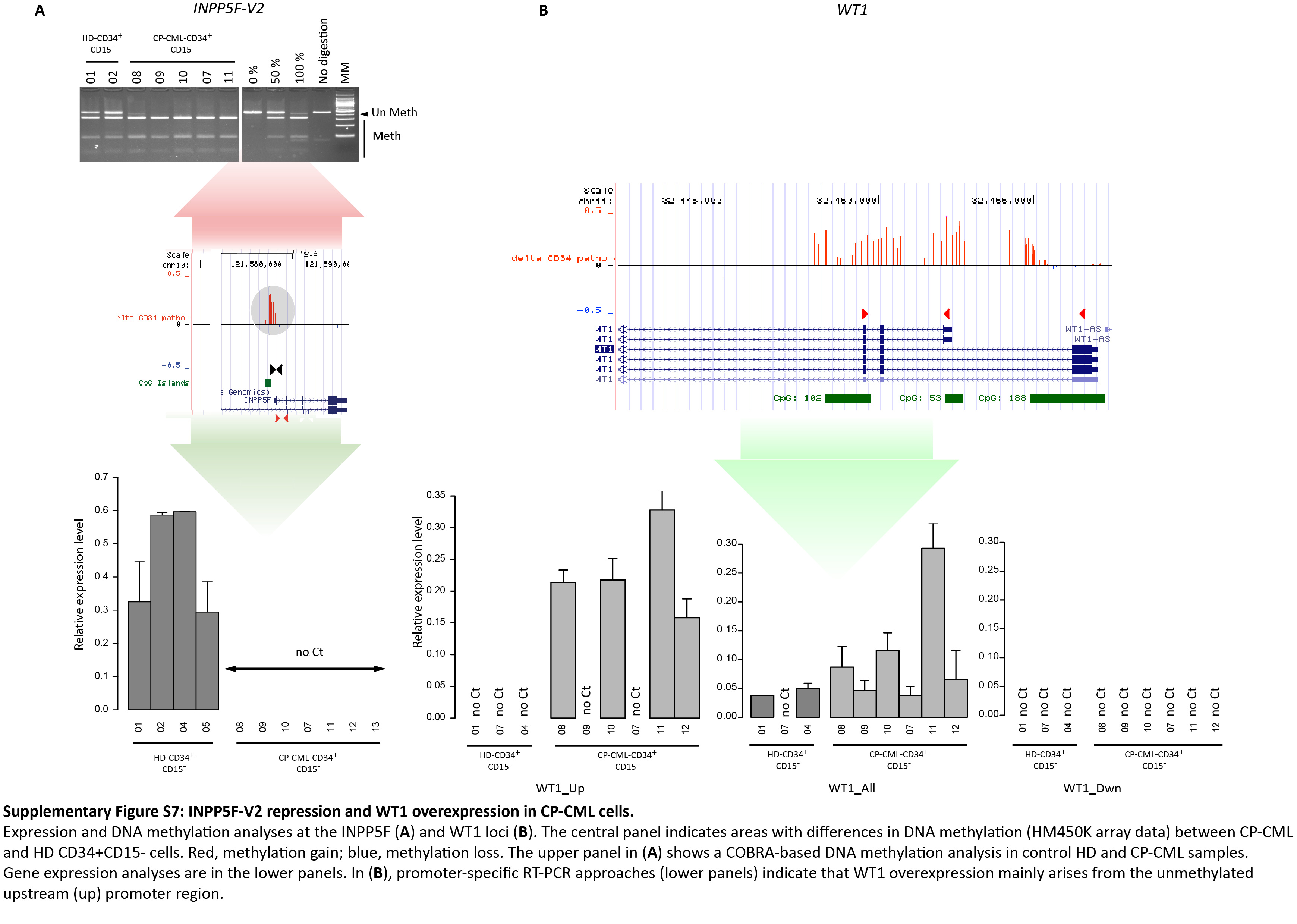

Supplement: Supplementary file 7 — Fig. S7. INPP5F‐V2 repression and WT1 overexpression in CP‐CML cells. [file MOL2-12-814-s007.jpg]
