# Supplementary material for: DNA methylation profiling reveals a pathological signature that contributes to transcriptional defects of CD34+ CD15− cells in early chronic‐phase chronic myeloid leukemia
Source: Mol Oncol. 2018 Apr 27;12(6):814–29. doi: 10.1002/1878-0261.12191 (PMC5983208; doi:10.1002/1878-0261.12191)
Supplement: Supplementary file 8 — Table S1. Details of the patients with CP‐CML. [file MOL2-12-814-s008.pdf]

Supplementary Table S1: Characteristics of the patients with CP-CML included in this study

#	Pt	Gender	Age at diagnosis	Sokal	HM450K	COBRA & bisulfite sequencing	RT-qPCR
1	CH G 053	F	74	High	×		
2	SA R 116	M	90	Intermediate	×		
3	PA A 117	M	48	Intermediate	×		
4	BA M 118	F	24	Low	×		
5	LA S 119	M	37	Intermediate	×		
6	FA A 120	M	52	Intermediate	×		
7	LE L 126	M	26	High		×	×
8	R S 005	M	44	Low		×	×
9	T D 022	M	46	Low		×	×
10	RO L 151	F	70	High		×	×
11	J J 016	M	36	Low		×	×
12	FO M 154	F	50	Low		×	×
13	NO D 159	M	64	Intermediate		×	×
