# Supplementary material for: DNA methylation profiling reveals a pathological signature that contributes to transcriptional defects of CD34+ CD15− cells in early chronic‐phase chronic myeloid leukemia
Source: Mol Oncol. 2018 Apr 27;12(6):814–29. doi: 10.1002/1878-0261.12191 (PMC5983208; doi:10.1002/1878-0261.12191)
Supplement: Supplementary file 9 — Table S2. (XLSX): Details of the primers used in this study. [file MOL2-12-814-s009.pdf]

Transcript Analysis								Methylation Analysis						
Locus	Primer name	Primer sequences 5' > 3'	Chr	Hg19 primer positions (5' > 3')		PCR Condition	PCR Amplification	Primer name	Primer sequences 5' > 3'	Hg19 primer positions (5' > 3')		PCR Condition	PCR Amplification	Restriction Enzyme for COBRA
Zap70	ZAP70_tr_F	CTTCATCGAGCAGGGCAAGC	2	98354484	98354503	SYBR Green Buffer 1X (Roche), [Primers F+R]= 0,5µM, cDNA (1/5)= 2µL	Lightcycler 480<br>95°C -> 2min<br>55 Cycles {95°C -> 12s ; 62°C -> 12s ; 72°C -> 12s}<br>Melting curve<br>40°C -> 10s	ZAP70_Bis_F	GTATTAGGTTTAGGAAGGTTTTGA	98340426	98340450	HotStar Taq Buffer 1X, [dNTP]f = 0,1mM, [$MgCl_2$]f = 1mM, [Primers F+R]f = 0,4µM, **HotStar Taq DNA polymerase(Qiagen)** = 1U, 1µL bisulfited DNA, qsp 20µL	95°C -> 15min<br>45 cycles { 95°C -> 30s ; 63°C -> 30s ; 72°C -> 20s}<br>72°C -> 10 min	Taq1
	ZAP70_tr_R	CTGCTCCACGGTCAGGAAGT		98355855	98355874			ZAP70_Bis_R	CTCTCACCTCCAACTTCCAC	98340889	98340909			
GAS2	GAS_tr_F	CTAGCCAGCAGACATGAAGCT	11	22696488	22696508	SYBR Green Buffer 1X (Roche), [Primers F+R]= 0,5µM, cDNA (1/5)= 2µL	Lightcycler 480<br>95°C -> 2min<br>55 Cycles {95°C -> 12s ; 62°C -> 12s ; 72°C -> 12s}<br>Melting curve<br>40°C -> 10s	GAS_Bis_F	AATTTGTGGGGATTAGTATATTTAG	22687553	22687578	HotStar Taq Buffer 1X, [dNTP]f = 0,1mM, [$MgCl_2$]f = 1mM, [Primers F+R]f = 0,4µM, **HotStar Taq DNA polymerase(Qiagen)** = 1U, 1µL bisulfited DNA, qsp 20µL	95°C -> 15min<br>45 cycles { 95°C -> 30s ; 58°C -> 30s ; 72°C -> 20s}<br>72°C -> 10 min	BstU1
	GAS_tr_R	CCGAGGGTGCACTGGTTTTG		22747864	22747883			GAS_Bis_R	AATATCAAAAACAATTATCTCCAAC	22687677	22687702			
PRAME	PRAME_tr_F	ATTTCCCCGGAGAAGGAAGA	22	22892256	22892275	SYBR Green Buffer 1X (Roche), [Primers F+R]= 0,5µM, cDNA (1/5)= 2µL	Lightcycler 480<br>95°C -> 2min<br>55 Cycles {95°C -> 12s ; 62°C -> 12s ; 72°C -> 12s}<br>Melting curve<br>40°C -> 10s	PRAME_Bis_F	TTTIGAGTTTGTAGAGGATTT	22901510	22901531	HotStar Taq Buffer 1X, [dNTP]f = 0,1mM, [$MgCl_2$]f = 1mM, [Primers F+R]f = 0,4µM, **HotStar Taq DNA polymerase(Qiagen)** = 1U, 1µL bisulfited DNA, qsp 20µL	95°C -> 15min<br>45 cycles { 95°C -> 30s ; 56°C -> 30s ; 72°C -> 20s}<br>72°C -> 10 min	HpyCH4IV
	PRAME_tr_R	GCTGGGACTCTGGGACAGAT		22890978	22890997			PRAME_Bis_R	CCTCTCCCCTTACCAAAA	22901785	22901803			
Inpp5fV2	IV2_tr_F	GACGTAATTTTTATGGCTTGGCTC	10	121579005	121579029	SYBR Green Buffer 1X (Roche), [Primers F+R]= 0,5µM, cDNA (1/5)= 2µL	Lightcycler 480<br>95°C -> 2min<br>55 Cycles {95°C -> 12s ; 62°C -> 12s ; 72°C -> 12s}<br>Melting curve<br>40°C -> 10s	IV2_Bis_F	GGTGTAGGTAGTTGTTTGAGTTT	121578261	121578284	HotStar Taq Buffer 1X, [dNTP]f = 0,1mM, [$MgCl_2$]f = 1mM, [Primers F+R]f = 0,4µM, **HotStar Taq DNA polymerase(Qiagen)** = 1U, 1µL bisulfited DNA, qsp 20µL	95°C -> 15min<br>5 Cycles {95°C -> 30s ; 61°C -> 30s ; 72°C -> 40s}<br>40 cycles { 95°C -> 30s ; 61°C -> 30s ; 72°C -> 20s}<br>72°C -> 10 min	Taq1
	IV2_tr_R	ACAGCACATCCACGTCTCTGT		121580375	121580395			IV2_Bis_R	AAAACAAAAACCCTTTAAATATTTC	121578682	121578707			
WT1	WT1_Up_tr_F	CGCTATTCGCAATCAGGGTTAC	11	32456247-32456262	32450166-32450160	SYBR Green Buffer 1X (Roche), [Primers F+R]= 0,5µM, cDNA (1/5)= 2µL	Lightcycler 480<br>95°C -> 2min<br>55 Cycles {95°C -> 12s ; 62°C -> 12s ; 72°C -> 12s}<br>Melting curve<br>40°C -> 10s							
	WT1_tr_2R	ATGGGATCCTCATGCTTGAATG		32450063	32450084									
WT1	WT1_tr_F	CCACAGCACAGGGTACGAGA	11	32421571	32421590	SYBR Green Buffer 1X (Roche), [Primers F+R]= 0,5µM, cDNA (1/5)= 2µL	Lightcycler 480<br>95°C -> 2min<br>55 Cycles {95°C -> 12s ; 62°C -> 12s ; 72°C -> 12s}<br>Melting curve<br>40°C -> 10s							
	WT1_tr_R	GCCTGGGTAAGCACACATGA		32417861	32417880									
WT1	WT1_Dwn_tr_F	GAGCAGGGCTCTGAGGATTG	11	32452125	32452144	SYBR Green Buffer 1X (Roche), [Primers F+R]= 0,5µM, cDNA (1/5)= 2µL	Lightcycler 480<br>95°C -> 2min<br>55 Cycles {95°C -> 12s ; 62°C -> 12s ; 72°C -> 12s}<br>Melting curve<br>40°C -> 10s							
	WT1_tr_2R	ATGGGATCCTCATGCTTGAATG		32450063	32450084									
SDHA	SDHA_tr_F	ACATCGGAACTGCGACTCA	5	240562	240581	SYBR Green Buffer 1X (Roche), [Primers F+R]= 0,5µM, cDNA (1/5)= 2µL	Lightcycler 480<br>95°C -> 2min<br>55 Cycles {95°C -> 12s ; 62°C -> 12s ; 72°C -> 12s}<br>Melting curve<br>40°C -> 10s							
	SDHA_tr_R	TTCTTGCAACACGCTTCCC		251139	251158									
GUSB	GUSB_tr_F	CATCGATGACATCACCGTCAC	7	65444409	65444429	SYBR Green Buffer 1X (Roche), [Primers F+R]= 0,5µM, cDNA (1/5)= 2µL	Lightcycler 480<br>95°C -> 2min<br>55 Cycles {95°C -> 12s ; 62°C -> 12s ; 72°C -> 12s}<br>Melting curve<br>40°C -> 10s							
	GUSB_tr_R	ACAGGTTACTGCCCTTGACA		65441148	65441167									
HMBS	HMBS_tr_F	AGAGTGATTCGCGTGGGTA	11	11895898	118959004	Tampon SYBR Green 1X, [Primers F+R]= 0,5µM, cDNA (1/5)= 2µL	Lightcycler 480<br>95°C -> 2min<br>55 Cycles {95°C -> 12s ; 62°C -> 12s ; 72°C -> 12s}<br>Melting curve<br>40°C -> 10s							
	HMBS_tr_R	CTTTCAATGTTGCCACCACAC		118959367	118959387									
