# Supplementary material for: DNA methylation profiling reveals a pathological signature that contributes to transcriptional defects of CD34+ CD15− cells in early chronic‐phase chronic myeloid leukemia
Source: Mol Oncol. 2018 Apr 27;12(6):814–29. doi: 10.1002/1878-0261.12191 (PMC5983208; doi:10.1002/1878-0261.12191)
Supplement: Supplementary file 11 — Table S4. Hotspots of DNA methylation alteration in CP‐CML cells. Genomic positions are according to hg19. [file MOL2-12-814-s011.docx]

**Supplementary Table S4**: Hotspots of DNA methylation alteration in CP-CML cells.

|  | chromosome | start | end | width | N probes | genomic features | Gene |
| --- | --- | --- | --- | --- | --- | --- | --- |
| Hypermethylated genomic areas  (>5 probes) in CP-CML CD34+ CD15- | chr19 | 52390810 | 52391789 | 980 | 14 | Prom | ZNF577,CTC-429C10.2 |
|  | chr6 | 32118204 | 32118457 | 254 | 14 | Prom | PRRT1 |
|  | chr5 | 78985425 | 78985900 | 476 | 10 | Prom | CMYA5 |
|  | chr6 | 24911204 | 24911553 | 350 | 9 | Prom | FAM65B |
|  | chr2 | 27665017 | 27665543 | 527 | 9 | Prom | KRTCAP3 |
|  | chr11 | 32451777 | 32452771 | 995 | 8 | Prom | WT1 |
|  | chr17 | 8702486 | 8702896 | 411 | 7 | Prom | MFSD6L |
|  | chr11 | 32449254 | 32450244 | 991 | 7 | gene_B | WT1 |
|  | chr6 | 31760593 | 31760796 | 204 | 7 | gene_B | VARS |
|  | chr19 | 13120555 | 13122567 | 2013 | 6 | gene_B | NFIX |
|  | chr19 | 58715251 | 58716032 | 782 | 6 | Enh | ZNF274 |
|  | chr9 | 133710056 | 133711284 | 1229 | 6 | Prom | ABL1 |
|  | chr6 | 28227068 | 28227127 | 60 | 6 | Prom | ZKSCAN4,NKAPL |
| Hypomethylated genomic areas  (>5 probes) in CP-CML CD34+ CD15- | chr6 | 31691354 | 31692795 | 1442 | 29 | Prom | C6orf25 |
|  | chr11 | 69468863 | 69469626 | 764 | 9 | gene_B | CCND1,ORAOV1 |
|  | chr6 | 30131189 | 30131613 | 425 | 9 | Prom | TRIM15 |
|  | chr20 | 3052115 | 3052345 | 231 | 8 | Prom | OXT |
|  | chr19 | 55549414 | 55549842 | 429 | 8 | Prom | GP6 |
|  | chr21 | 43823749 | 43824262 | 514 | 7 | Prom | UBASH3A |
|  | chr17 | 77895596 | 77896584 | 989 | 7 | gene_B | RP11-353N14.5,RP11-353N14.4 |
|  | chr7 | 16505094 | 16505664 | 571 | 7 | Prom | SOSTDC1 |
|  | chr7 | 56160687 | 56160911 | 225 | 7 | Prom | PHKG1 |
|  | chr7 | 65419185 | 65419288 | 104 | 7 | gene_B | VKORC1L1 |
|  | chr6 | 32014476 | 32014739 | 264 | 7 | Prom | TNXB |
|  | chr11 | 368366 | 368573 | 208 | 6 | Enh |  |
|  | chr10 | 135202522 | 135203200 | 679 | 6 | gene_B | PAOX |
|  | chr6 | 31762353 | 31762630 | 278 | 6 | Prom | VARS |
|  | chr6 | 32135715 | 32136052 | 338 | 6 | Prom | EGFL8 |
|  | chr6 | 32805398 | 32805692 | 295 | 6 | Prom | TAP2 |
|  | chr6 | 167536056 | 167536521 | 466 | 6 | Prom | CCR6 |
|  | chr4 | 99851003 | 99851211 | 209 | 6 | Prom | EIF4E,AC019131.1,RP11-571L19.7 |
|  | chr1 | 42384310 | 42384647 | 338 | 6 | Prom | HIVEP3 |
|  | chr1 | 223566669 | 223567173 | 505 | 6 | Prom | C1orf65 |
|  |  |  |  |  |  |  |  |
| Hypermethylated genomic areas  (>5 probes) in CP-CML CD34- CD15+ | chr6 | 24910562 | 24911615 | 1054 | 14 | Prom | FAM65B |
|  | chr19 | 52390810 | 52391367 | 558 | 10 | Prom | ZNF577,CTC-429C10.2 |
|  | chr11 | 32449254 | 32450692 | 1439 | 10 | gene_B | WT1 |
|  | chr16 | 8806663 | 8807043 | 381 | 9 | Prom | ABAT |
|  | chr6 | 30095174 | 30095277 | 104 | 9 | inter_G |  |
|  | chr5 | 78985432 | 78985900 | 469 | 9 | Prom | CMYA5 |
|  | chr2 | 27665017 | 27665543 | 527 | 9 | Prom | KRTCAP3 |
|  | chr17 | 8702369 | 8702896 | 528 | 8 | Prom | MFSD6L |
|  | chr11 | 32451777 | 32452771 | 995 | 8 | Prom | WT1 |
|  | chr17 | 7832680 | 7833237 | 558 | 7 | Prom | KCNAB3,RP11-1099M24.7 |
|  | chr3 | 113160183 | 113160623 | 441 | 7 | Prom | WDR52 |
|  | chr19 | 58715251 | 58716032 | 782 | 6 | Enh | ZNF274 |
|  | chr10 | 121577971 | 121578846 | 876 | 6 | Prom | INPP5F |
|  | chr9 | 133710056 | 133711284 | 1229 | 6 | Prom | ABL1 |
|  | chr8 | 87081770 | 87082045 | 276 | 6 | Prom | PSKH2 |
|  | chr6 | 28227068 | 28227127 | 60 | 6 | Prom | ZKSCAN4,NKAPL |
|  | chr6 | 49681178 | 49681334 | 157 | 6 | Prom | CRISP2 |
|  | chr5 | 140801354 | 140802804 | 1451 | 6 | Prom | PCDHGA11 |
|  | chr2 | 219737392 | 219738732 | 1341 | 6 | gene_B | WNT6 |
| Hypomethylated genomic areas  (>5 probes) in CP-CML CD34- CD15+ | chr6 | 31691354 | 31691597 | 244 | 10 | Prom | C6orf25 |
|  | chr6 | 31691696 | 31692080 | 385 | 9 | Prom | C6orf25 |
|  | chr20 | 3052115 | 3052345 | 231 | 8 | Prom | OXT |
|  | chr19 | 55549414 | 55549842 | 429 | 8 | Prom | GP6 |
|  | chr6 | 30131189 | 30131570 | 382 | 8 | Prom | TRIM15 |
|  | chr17 | 79004947 | 79006087 | 1141 | 7 | gene_B | BAIAP2-AS1 |
|  | chr6 | 32014476 | 32014739 | 264 | 7 | Prom | TNXB |
|  | chr1 | 42384310 | 42384647 | 338 | 6 | Prom | HIVEP3 |
